# The Association Between HbA1c Level and the Outcomes of Rotator Cuff Repair Surgery in Patients Managed at a Tertiary Center: A Retrospective Cohort Study

**DOI:** 10.7759/cureus.94387

**Published:** 2025-10-12

**Authors:** Ahmed AlHussain, Abdullah M Alghamdi, Talal H BinMushayt, Abdulaziz G Alotaibi, Omar F Alsalem, Ziad A Aljaafri

**Affiliations:** 1 Orthopaedic Surgery, King Abdulaziz Medical City, Riyadh, SAU; 2 College of Medicine, King Saud Bin Abdulaziz University for Health Sciences, Riyadh, SAU

**Keywords:** diabetes mellitus, glycated hemoglobin (hba1c), glycemic control, postoperative outcomes, rotator cuff repair surgery

## Abstract

Background

Rotator cuff tears are a leading cause of shoulder dysfunction and often require surgical repair. Diabetes mellitus can impair tendon healing through chronic inflammation and poor glycemic control, reflected by elevated glycated hemoglobin (HbA1c) levels. This study aimed to explore the association between HbA1c levels and the outcomes of rotator cuff repair surgery in patients managed at a tertiary center.

Methodology

This retrospective cohort study was conducted at King Abdulaziz Medical City, Riyadh, Saudi Arabia, from February 2020 to February 2025. Data were collected on demographics, pre- and postoperative details, outcomes, and comorbidities. Data were analyzed using Fisher’s exact test, Spearman’s correlation, independent-sample t-tests, and descriptive statistics, conducted using SPSS version 29.0.0 (IBM Corp., Armonk, NY, USA).

Results

Of the 79 eligible patients, 38 were excluded due to incomplete HbA1c data, and 41 were analyzed. Most patients were female (28; 68.3%) with a mean age of 57.1 ± 7.6 years (range = 39-69 years). The average body mass index was 30.2 ± 4.3 kg/m², with 18 (43.9%) patients classed as overweight and 19 (46.3%) obese. Common comorbidities included dyslipidemia (32; 78.0%), hypertension (23; 56.1%), and diabetes (21; 51.2%). Preoperative HbA1c showed no correlation with functional improvements in abduction (r = 0.132, p = 0.455), forward flexion (r = −0.082, p = 0.646), external rotation (r = 0.292, p = 0.187), or return to work (r = 0.008, p = 0.961). Complication rates were similar between controlled (6; 66.7%) and uncontrolled (3; 33.3%) HbA1c groups (p = 0.707). Hypertension (B = 1.327, p = 0.032) and liver disease (B = 3.913, p = 0.009) predicted higher postoperative HbA1c, while other comorbidities showed no significant associations.

Conclusions

Preoperative HbA1c was not associated with postoperative functional outcomes or complications. However, certain comorbidities were linked to higher postoperative HbA1c, suggesting that overall health conditions may influence recovery more than glycemic control alone. These findings highlight the importance of comprehensive perioperative assessment and tailored management strategies to optimize outcomes in rotator cuff repair patients.

## Introduction

Rotator cuff tears are among the most common causes of shoulder pain and dysfunction, and surgical intervention is often required when conservative management fails [1]. Rotator cuff repair (RCR) is associated with considerable success in restoring shoulder function, reducing pain, and improving quality of life [2]. However, despite advancements in arthroscopic techniques and rehabilitation protocols, a subset of patients continues to experience suboptimal outcomes, including poor tendon healing, re-tears, and functional limitations. This variability of outcomes has drawn attention to systemic factors that may impair tendon-to-bone healing, with diabetes mellitus (DM) emerging as a critical and under-recognized factor [3].

DM, particularly type 2, is a global public health concern that impacts all systems of the body, including musculoskeletal tissue, through various mechanisms [4]. These mechanisms include microvascular compromise, chronic inflammation, and impaired collagen remodeling [5]. Glycated hemoglobin (HbA1c) is a validated surrogate marker, routinely used to assess long-term glycemic control, DM severity, and management efficacy. Elevated HbA1c levels have been associated with delayed wound healing and increased risk of postoperative infections [6]. Poorer orthopedic surgical outcomes in procedures such as total joint arthroplasty and spinal fusion have also been reported [7]. However, the precise influence of preoperative glycemic status, as reflected by HbA1c levels, on the outcome of RCR surgery remains inadequately defined.

Recent literature suggests that hyperglycemia may adversely affect tendon biology by reducing fibroblast activity, impairing collagen cross-linking, and ultimately compromising the healing interface between tendon and bone [8]. These biochemical and cellular changes may result in weaker repairs, bringing a higher risk of structural failure. Moreover, poorly controlled DM is associated with greater postoperative complication rates, including adhesive capsulitis, infection, and longer rehabilitation timelines [9]. In contrast, patients with well-controlled DM may experience comparable outcomes to their non-diabetic counterparts, underscoring the potential modifiability of this risk factor.

Therefore, understanding the association between HbA1c levels and RCR outcomes is essential. This is especially true in tertiary care settings, where complex and comorbid patient populations are common. The identification of a clinically relevant HbA1c threshold that correlates with adverse outcomes would be an important therapeutic advance. Such a threshold could inform preoperative risk stratification, enabling the optimization of perioperative management and facilitating surgical success. Additionally, this strategy could support the implementation of tailored perioperative protocols, including multidisciplinary glycemic optimization before elective RCR procedures.

Despite the biological plausibility and available early observational data, the current evidence for this approach remains inconclusive. While some studies have reported significant associations, others have failed to demonstrate a correlation between HbA1c and postoperative outcomes [10,11]. This ambiguity highlights the need for further research, particularly in Middle Eastern populations. The overall prevalence of DM is high in this region (14.6%), with Kuwait, Saudi Arabia, and the United Arab Emirates having the highest rates (>21%), and various culturally specific factors may influence disease management and surgical care [12]. Although DM is highly prevalent in these regions, no study has focused on examining the association between preoperative HbA1c levels and the outcomes of RCR.

This retrospective cohort study aimed to investigate the association between preoperative HbA1c levels and postoperative outcomes of RCR surgery among patients treated at a tertiary care center in Saudi Arabia. The primary objective of this study was to evaluate the association between preoperative HbA1c levels and postoperative functional outcomes following RCR and to assess the relationship between preoperative HbA1c levels and postoperative complications.

## Materials and methods

Data sources

A retrospective study was conducted in King Abdulaziz Medical City (KAMC), Health Affairs, Ministry of National Guard (MNGHA), Riyadh, Saudi Arabia, from February 2020 to February 2025. The study was reviewed and approved by the Institutional Review Board (IRB) of King Abdullah International Medical Research Center (KAIMRC). No identifiers were collected to preserve patients’ confidentiality and anonymity. All data, both soft and hard copies, were maintained within MNGHA premises and were accessible only to the research team.

Study selection criteria

No formal sample size calculation was required, as all patients over the age of 18 years diagnosed with a torn rotator cuff and who underwent RCR surgery during the study period were considered. Inclusion criteria were patients aged 18 years or older with a minimum follow-up of 12 months, had a recorded and assessed pre- and postoperative range of motion (ROM) taken during their follow-up clinic visits, those who followed postoperative protocol, in which patients were immobilized in an arm sling for six weeks with allowance for elbow and hand active ROM exercises, followed by initiation of supervised physiotherapy sessions to restore shoulder mobility and strength, and a record of pre- and postoperative HbA1c taken within six months, respectively. Patients who underwent emergency or revision RCR surgery, pediatric patients, or patients with osteoarthritis of Kellgren-Lawrence grade 2 or higher in the operated joint, follow-up under 12 months, or the absence of electronic medical records were excluded from the study.

Upon initial chart review, we identified a total of 79 patients who had undergone RCR. We excluded 38 patients for incomplete follow-up (<12 months), lack of pre- or postoperative HbA1C levels recorded within six months (n = 7), incomplete medical records (n = 4), and pediatric status (n = 3). A total of 41 patients remained and were included in the analysis.

Variable definitions

The collected data were transferred into an Excel (Microsoft Corp., Redmond, WA, USA) sheet to be cleaned and reviewed for data selection and analysis. The collected variables included demographics (age, gender, body mass index (BMI)), smoking status, comorbid conditions (DM, hypertension, thyroid disease, rheumatoid arthritis, chronic kidney disease, acute kidney disease, dyslipidemia, liver disease, and history of cancer), pre- and postoperative HbA1c levels, postoperative complications, surgical approach (arthroscopic, open, or mini-open), reoperation, pre- and postoperative ROM, preoperative length of stay (LOS), postoperative LOS, total hospital stay, and prior shoulder surgeries. Controlled HbA1c was defined as <7%; this cutoff was based on the American Diabetes Association Standards of Medical Care in Diabetes 2024, which recommends maintaining HbA1c below 7% for most non-pregnant adults to minimize the risk of microvascular complications. Uncontrolled HbA1c was defined as ≥7%. While we acknowledge that operative tolerances in surgical practice may extend slightly higher (up to 7.5%-8%) without markedly increasing perioperative risk, we used the <7% threshold to ensure consistency with widely accepted medical definitions and facilitate comparability with prior studies [13].

Statistical analysis

Comprehensive statistical analysis was conducted on the dataset, encompassing both descriptive and inferential methodologies. A descriptive analysis was conducted to summarize the demographic characteristics of the participants, including age, gender, and other features. Fisher’s exact test was used to examine the association between categorical variables. Subsequently, Spearman’s test was used to identify correlations between HbA1c and functional outcomes. Independent-samples t-tests were used to examine the difference between continuous variables. All statistical analyses were performed using SPSS Software, version 29.0.0 (IBM Corp., Armonk, NY, USA).

## Results

Our study included 79 patients, of whom 38 were excluded due to incomplete data. Of the 41 included patients, the majority were female (28; 68.3%), with males accounting for 31.7% (n = 13) of the cohort. The mean patient age was 57.1 years (SD = 7.6 years; range = 39-69 years). In terms of BMI, most patients were either obese (19; 46.3%) or overweight (18; 43.9%), with only four (9.8%) patients falling within the normal BMI range. The mean BMI was 30.2 kg/m² (SD = 4.3), with values ranging from 22.3 kg/m² to 39.9 kg/m² (Table 1).

**Table 1:** Sociodemographic parameters of patients (n = 41).

		N (%)
Gender	Female	28 (68.3%)
	Male	13 (31.7%)
Age (years)	Mean (SD)	57.1 (7.6)
	Range	39–69
Body mass index	Normal (18.5–24.9 kg/m^2^)	4 (9.8%)
	Overweight (25.0–29.9 kg/m^2^)	18 (43.9%)
	Obese (≥30 kg/m^2^)	19 (46.3%)
	Mean (SD)	30.2 (4.3)
	Range	22.3–39.9

**Figure 1:**
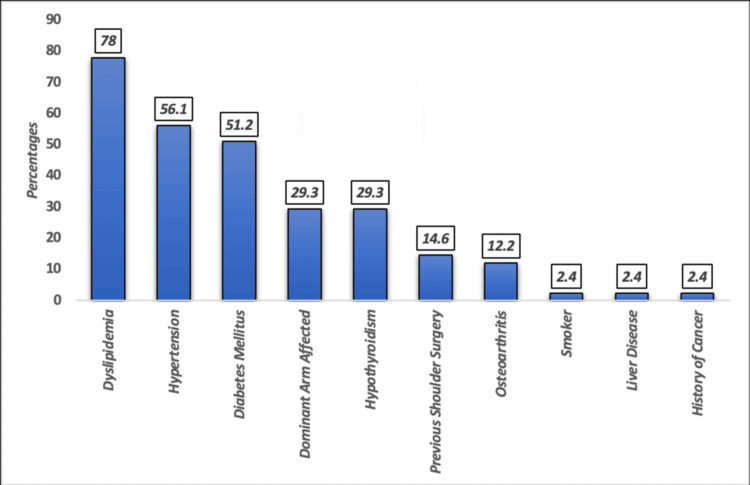
Distribution of different comorbidities in rotator cuff surgery repair patients (n = 41).

Figure 1 shows the distribution of comorbidities among patients who had undergone RCR surgery (n = 41). The most prevalent condition was dyslipidemia (78%), followed by hypertension (56.1%) and DM (51.2%). Both dominant arm involvement and hypothyroidism were noted in 29.3% of cases. Less common conditions included previous shoulder surgery (14.6%) and osteoarthritis (12.2%). Rare comorbidities, each present in only 2.4% of the cohort, included smoking, liver disease, and a history of cancer.

Table 2 shows the correlation between the preoperative HbA1c levels and postoperative functional improvements in patients who underwent RCR (n = 41). The correlation between preoperative HbA1c level and improvement in abduction range was statistically non-significant (r = 0.132, p = 0.455); the correlation with forward flexion was also non-significant (r = −0.082, p = 0.646). The improvement in external rotation did not correlate with preoperative HbA1c level (r = 0.292, p = 0.187), nor did time to return to work (r = 0.008, p = 0.961).

**Table 2:** Correlation between preoperative HbA1c levels and postoperative functional outcomes following rotator cuff repair (n = 41). HbA1c: glycated hemoglobin

		Preoperative HbA1c level
Improvement in abduction range	Correlation, r (95% CI)	0.132 (-0.225 to 0.459)
	Sig.	0.455
Improvement in forward flexion	Correlation, r (95% CI)	−0.082 (-0.417 to 0.273)
	Sig.	0.646
Improvement in external rotation	Correlation, r (95% CI)	0.292 (-0.161 to 0.643)
	Sig.	0.187
Time to return to work	Correlation, r (95% CI)	0.008 (-0.309 to 0.323)
	Sig.	0.961

Table 3 shows the association between preoperative HbA1c levels and postoperative complications following RCR. A total of nine patients reported postoperative complications, of whom six (66.7%) had controlled HbA1c, versus three (33.3%) in the uncontrolled HbA1c group (p = 0.707). Of the patients reporting pain or stiffness, two (66.7%) had controlled and one (33.3%) had uncontrolled HbA1c (p = 1.000). Hypertrophic scars were seen in two (100%) controlled patients, with none in the uncontrolled group (p = 0.495). Cases of delayed wound healing occurred in six (66.7%) controlled and three (33.3%) uncontrolled patients (p = 0.707). Reoperation and re-tear each occurred in one (100.0%) patient with uncontrolled HbA1c. Golfer’s elbow was reported in one (100.0%) patient with controlled HbA1c. Postoperative LOS was similar across both groups (median = 1 day; IQR = 1; p = 0.889). No statistically significant associations were observed.

**Table 3:** Associations between preoperative HbA1c levels and postoperative complications following rotator cuff repair surgery. HbA1c: glycated hemoglobin

		Hyperglycemia		Chi-square value	P-value
		Controlled HbA1c (<7%), N (%)	Uncontrolled HbA1c (≥7%), N (%)		
Presence of any complications	No	17 (54.8%)	14 (45.2%)	0.399	0.707
	Yes	6 (66.7%)	3 (33.3%)		
Pain/Stiffness	No	20 (54.1 %)	17 (45.9 %)	0.178	1.000
	Yes	2 (66.7 %)	1 (33.3 %)		
Hypertrophic scar	No	21 (53.8 %)	18 (46.2 %)	1.645	0.495
	Yes	2 (100 %)	0 (0 %)		
Delayed healing	No	17 (54.8%)	14 (45.2%)	0.399	0.707
	Yes	6 (66.7%)	3 (33.3%)		
Reoperation	No	23 (57.5%)	17 (42.5%)	1.310	0.439
	Yes	0 (0.0%)	1 (100.0%)		
Golfer elbow	No	22 (55.0%)	18 (45.0%)	0.802	1.000
	Yes	1 (100.0%)	0 (0.0%)		
Retear	No	23 (57.5%)	17 (42.5%)	1.310	0.493
	Yes	0 (0.0%)	1 (100.0%)		
Postoperative length of stay	Median (IQR)	1 (1)	1 (1)	0.023	0.889

Table 4 shows correlations between patient comorbidities and postoperative HbA1c levels following RCR. Among the examined variables, hypertension showed a significant positive association, with these patients exhibiting 1.33% higher postoperative HbA1c on average compared to non-hypertensive patients (B = 1.327, 95% CI = 0.126-2.528, p = 0.032). Similarly, liver disease was significantly associated with elevated HbA1c, demonstrating a mean increase of 3.91% (B = 3.913, 95% CI = 1.065-6.762, p = 0.009). Other comorbidities, including BMI, smoking status, hypothyroidism, dyslipidemia, osteoarthritis, history of cancer, and previous surgery, showed no statistically significant relationships (all p > 0.05). Thus, hypertension and liver disease independently contribute to higher postoperative HbA1c levels, underscoring their clinical relevance in perioperative metabolic control.

**Table 4:** Correlation of comorbidities in patients undergoing rotator cuff repair surgery with postoperative HbA1c levels. BMI: body mass index; HbA1c: glycated hemoglobin

	B	SE	t	Sig.	95% CI (lower limit–upper limit)
BMI	-0.025	0.058	-0.424	0.675	-0.144–0.095
Comorbidity	-0.428	0.873	-0.491	0.628	-2.216–1.359
Smoker	-1.095	1.393	-0.786	0.439	-3.949–1.760
Hypertension	1.327	0.586	2.262	0.032	0.126–2.528
Hypothyroidism	-0.070	0.505	-0.138	0.891	-1.104–0.964
Dyslipidemia	0.099	0.732	0.135	0.893	-1.401–1.599
Osteoarthritis	-0.695	0.785	-0.886	0.383	-2.303–0.912
Liver disease	3.913	1.391	2.814	0.009	1.065–6.762
History of cancer	2.806	1.625	1.727	0.095	-0.522–6.135
Previous surgery	-0.517	0.596	-0.867	0.394	-1.739–0.705

Table 5 shows the impact of different comorbidities on perioperative changes in HbA1c among patients undergoing RCR. Patients with hypertension showed a slight mean increase in HbA1c (+0.11), compared to a mild decrease in non-hypertensive patients (-0.06; p = 0.290). Similarly, dyslipidemia was associated with a small rise (+0.07), while non-dyslipidemic patients showed a slight decline (-0.07; p = 0.479). Hypothyroidism produced a modest increase (+0.20) versus a minor reduction (-0.03) in euthyroid patients (p = 0.171). In contrast, those who had undergone previous surgeries experienced a reduction in HbA1c (-0.18) compared to an increase (+0.07) in patients without (p = 0.237). Osteoarthritis was linked to a marginal rise (+0.18) versus stability (-0.01) in unaffected patients (p = 0.435).

**Table 5:** Impact of different comorbidities on perioperative changes in HbA1c levels in rotator cuff surgery patients. Independent-samples t-test.

		Perioperative ΔHbA1c	t-value	P-value
Hypertension	No	-0.06	-1.454	0.290
	Yes	0.11		
Dyslipidemia	No	-0.07	-1.256	0.479
	Yes	0.07		
Hypothyroidism	No	-0.03	1.286	0.171
	Yes	0.20		
Previous surgery	No	0.07	0.660	0.237
	Yes	-0.18		
Osteoarthritis	No	-0.01	0.211	0.435
	Yes	0.18		

## Discussion

This retrospective study aimed to assess whether preoperative HbA1c levels influence postoperative outcomes in RCR patients treated at a tertiary care center. The main finding was that preoperative HbA1c showed no correlation with functional improvements in abduction, forward flexion, external rotation, or return to work. A study by Sambandam et al. (2015) showed that rotator cuff tears are a common cause of shoulder dysfunction, often requiring surgical repair [14]. Vartanian et al. (2025) showed that RCR surgery generally improves function, outcomes such as ROM, and the occurrence of postoperative complications vary, especially in patients with comorbidities such as DM and hypertension [15]. Poor glycemic control is reflected by elevated HbA1c (>7%) and may impair tendon healing and increase rates of complications. However, the existing evidence on the impact of HbA1c on RCR outcomes is inconclusive, particularly in Middle Eastern populations where the prevalence of DM is very high.

Our results show that in a predominantly female (68.3%) and middle‑aged (mean = 57 ±7.6 years) cohort, there was a high prevalence of metabolic comorbidities, including obesity/overweight (~90%), dyslipidemia (78%), hypertension (56.1%), and diabetes (51.2%). This aligns with a previous study by Kim et al. (2020) on the demographics of rotator cuff disease, which disproportionately affects older adults and those with cardiometabolic risk [16]. Another study by Giri et al. (2023) reported that cardiometabolic risk factors such as DM, hypertension, and hyperlipidemia are associated with both rotator cuff disease and postoperative complications [17].

Notably, our study did not find any significant correlation between preoperative HbA1c levels and postoperative improvements in outcomes, including shoulder abduction, forward flexion, external rotation, or time to return to work. Preoperative HbA1c showed no significant association with any measured postoperative functional outcomes. In contrast, Liang et al. (2025) previously reported that preoperative hyperglycemia and HbA1c level are associated with reduced shoulder motion, increased pain, and higher postoperative retear risk after arthroscopic RCR [18]. Effective glycemic control may help reduce re-tear rates. In the current study, there was a single case of re-tear, which occurred in the uncontrolled HbA1c group. However, a statistical association was not noted. Well-maintained glycemic control in the postoperative period may contribute to rotator cuff healing. Takahashi et al. (2022) also demonstrated worse ROM and functional scores in patients with poor glycemic control [19]. However, such studies often focus on comparisons of DM versus non‑DM patients, or use broader clinical scoring systems (e.g., Constant or UCLA scores). In contrast, our dataset captured specific ROM improvements.

Our results found no statistically significant association between preoperative HbA1c levels and the postoperative complications after RCR. While there was a higher proportion of complications in the controlled HbA1c group, including pain/stiffness, delayed healing, and hypertrophic scarring, these differences were not statistically significant (p > 0.05). Notably, reoperation and re-tear were exclusively observed in the uncontrolled group, hinting at a potential, albeit inconclusive, trend. Only one reoperation occurred in an uncontrolled HbA1c patient. Another large-scale database study by Takahashi et al. (2022) showed significantly worse outcomes in the high HbA1c group in terms of forward flexion (159.5° vs. 167.3°, p = 0.013), Constant score (90.1 vs. 94.9, p = 0.033), and UCLA score (30.6 vs. 32.6, p = 0.037). They also reported a modestly increased infection risk at higher HbA1c thresholds (e.g., >8%), but no increase in revision surgery or stiffness independent of glycemic control [19]. Another meta-analysis by Alhussain et al. (2025) linked elevated HbA1c levels to increased postoperative complications such as stiffness and poor function after RCR [20]. One national database study by Cancienne et al. (2019) demonstrated an increased infection rate, from ~0.3% to ~0.84%, when perioperative HbA1c exceeded 8%, while revision and lysis of adhesions/manipulation under anesthesia rates remained unaffected [21]. Thus, mild HbA1c elevations, as seen in many patients in our sample, may have a limited impact on complications.

Our regression model revealed two comorbidities, hypertension and liver disease, with statistically significant associations with postoperative HbA1c levels. This shows the clinical importance of these conditions in terms of postoperative HbA1c levels and outcomes for patients undergoing RCR. Hypertension was associated with an average 1.33% increase in postoperative HbA1c (B = 1.327, 95% CI = 0.126-2.528, p = 0.032). Chronic hypertension is closely linked to insulin resistance, vascular dysfunction, and systemic inflammation, all of which disrupt glucose metabolism. This sustained hyperglycemic environment may impair tendon-to-bone healing, increasing the risk of suboptimal repair integrity, delayed functional recovery, and impaired ROM. Similarly, Loyst et al. (2023) reported a direct link between hypertension and poor outcomes following RCR, identifying the condition as a risk factor for various postoperative complications [22].

Similarly, liver disease exerted an even stronger effect, with HbA1c rising by 3.91% (B = 3.913, 95% CI = 1.065-6.762, p = 0.009). Given the liver’s central role in glycogen storage, gluconeogenesis, and insulin sensitivity, hepatic dysfunction may create a metabolic environment in which postoperative glycemic control becomes particularly challenging. Subsequently, elevated glucose levels may alter collagen cross-linking and fibroblast activity, weaken the biological foundations of tendon healing, and increase the risk of surgical failure after RCR. Kinnard et al. (2024) showed that patients with liver disease have an increased risk of complications following orthopedic surgery and an increased rate of failure for surgical outcomes [23]. Meanwhile, Kandikattu et al. (2025) reported that patients with comorbidities such as metabolic syndrome, hypertension, and liver dysfunction have prolonged hospital stays, a higher incidence of reinjury, and increased instances of rehospitalization after RCR [24]. Thus, accumulated comorbidities may impair glycemic recovery after surgical stress. These findings align with the study by Smiley et al. (2006), which suggested that systemic disease burden undermines perioperative glucose control [25]. However, most prior studies have focused on preoperative values rather than postoperative HbA1c trends.

Limitations

There are several important limitations of this study that should be acknowledged. First, the sample size was relatively small (n = 41), limiting the statistical power and reducing the ability to detect subtle but clinically meaningful associations. Second, the study was retrospective in nature, introducing inherent risks of selection bias and incomplete data capture. Third, our dataset did not include the exact timing of the postoperative HbA1c measurement, which prevented us from distinguishing whether the elevations occurred during the critical tendon healing window (e.g., 3-6 months). This limits the generalizability and interpretability of the postoperative HbA1c as a prognostic marker. Furthermore, the integrity of the tendon was not assessed with postoperative imaging, preventing any assessment of correlations between glycemic status and re-tear rates. Finally, the presence of multiple comorbidities in patients complicates causal inference, as hypertension and liver disease may elevate HbA1c and indirectly worsen the outcomes, but residual confounding cannot be excluded. Future prospective studies with larger cohorts, standardized follow-up intervals, patient-reported outcome measures, and imaging-based tendon integrity assessments are recommended.

Implications and future directions

Preoperative HbA1c may not directly affect short-term outcomes after RCR surgery, but postoperative increases in HbA1c during the healing phase may compromise tendon integrity. Patients with diabetes and hypertension are particularly vulnerable to poor postoperative glycemic control. This underscores the importance of closely monitoring, maintaining, or improving stable HbA1c levels during the critical 3-6 months post-surgery.

## Conclusions

Our results indicate that preoperative HbA1c does not significantly correlate with improvements in ROM across abduction, forward flexion, or external rotation. Similarly, HbA1c levels showed no significant association with postoperative complications, including pain, stiffness, delayed healing, or re-tear. Hypertension and liver disease are significant contributors to higher postoperative HbA1c levels, which may impair recovery of ROM across abduction, forward flexion, or external rotation. Thus, HbA1c alone did not emerge as a strong predictor of functional recovery or complication risk following surgery. However, given the limited sample size, these findings may not fully reflect the associations that could be observed in a large cohort study.
